# Analyzing the impact of loyalty card programs on customer behavior: insights from the Albanian market

**DOI:** 10.12688/f1000research.138185.3

**Published:** 2023-11-21

**Authors:** Ervin Myftaraj, Brunela Trebicka

**Affiliations:** 1 Department of Marketing, Universiteti Aleksander Moisiu Durres, Durrës, Durrës County, Albania; 2 Applied Statistics and Informatics, Universiteti Aleksander Moisiu Durres, Durrës, Durrës County, Albania

**Keywords:** loyalty card programs, customer behavior, awareness, satisfaction, loyalty, customized benefits

## Abstract

**Background:**

Loyalty card programs have become prevalent in the retail industry, offering various benefits and rewards to customers. This study aims to analyze the impact of loyalty card programs on customer behavior in the Albanian market.

**Methods:**

A survey was conducted among a diverse sample of customers from different retail sectors in Albania. The survey collected data on awareness, satisfaction, loyalty, and customized benefits. The survey was distributed between March and May 2023, and responses were collected electronically. The survey responses were analyzed to address the research questions and test the hypotheses.

**Results:**

The findings indicate a significant level of awareness among respondents regarding loyalty card programs. Higher satisfaction with specific benefits, such as discounts and accumulating points, is associated with higher levels of customer loyalty. Customers who reported higher frequencies of loyalty card usage demonstrated greater loyalty. Customized benefits, particularly discounts and accumulating points, significantly influenced customer satisfaction. However, other benefit categories did not show statistically significant associations with loyalty.

**Conclusion:**

The study highlights the importance of promoting loyalty card programs to enhance awareness among customers. Improving and optimizing loyalty card benefits based on customer feedback is recommended. Encouraging frequent usage of loyalty cards and personalizing benefits to align with customer preferences are key strategies to foster customer loyalty. The findings provide valuable insights for practitioners in the retail sector to enhance customer engagement, satisfaction, and loyalty.

## Introduction

### Background

In today’s competitive retail market, businesses face the ongoing challenge of attracting and retaining customers. Loyalty card programs have emerged as popular marketing tools that offer a range of benefits and rewards to participating customers. These programs are designed to foster customer loyalty, increase customer retention, and drive repeat purchases ( Schuhmann and Kwortnik, 2019). The impact of loyalty card programs on customer behavior has been extensively studied across different markets, providing valuable insights for both practitioners and researchers ( Smith and Johnson, 2019).

Research conducted by Smith and Johnson (2019) highlights the prevalence of loyalty card programs in the retail industry, with supermarkets and chain stores being prominent players in offering such programs. Smith and Johnson (2021) state that loyalty card program typically provide customers with a variety of benefits, including discounts, accumulating points, exclusive offers, personalized recommendations, and a faster checkout process. The underlying assumption is that by providing these incentives, businesses can enhance customer satisfaction and cultivate long-term loyalty.

Studies have shown that loyalty card programs can have a significant impact on customer behavior and satisfaction. For example, a study by Ping-Lung *et al.* (2017) found that customers who actively participated in a loyalty card program exhibited higher levels of satisfaction and loyalty compared to non-participants. Another study by Johnson *et al.* (2018) demonstrated that loyalty card benefits such as personalized recommendations and exclusive offers were positively associated with customer satisfaction and loyalty.

Moreover, loyalty card programs have been found to influence various aspects of customer behavior, including shopping frequency and spending patterns. In their research on the impact of loyalty card programs, Brown and Smith (2020) discovered that customers who were engaged in a loyalty program visited the store more frequently and spent more on their purchases compared to non-participants. This suggests that loyalty card programs can effectively encourage repeat purchases and increase customer engagement.

The effectiveness of loyalty card programs is further supported by a study conducted by Nguyen and Nguyen (2019), which revealed that customers who were satisfied with the benefits provided by loyalty cards demonstrated higher levels of loyalty and were more likely to recommend the store to others. This highlights the positive relationship between customer satisfaction with loyalty card programs and their likelihood to exhibit loyal behaviors and engage in positive word-of-mouth recommendations.

Furthermore, customization and personalization have been recognized as key factors in loyalty card program success. Research by Anderson *et al.* (2018) emphasized the importance of tailoring loyalty card benefits to meet customer preferences and needs. By customizing benefits such as discounts and rewards based on individual customer data and preferences, businesses can enhance customer satisfaction and loyalty.

Existing studies on loyalty card programs, while providing a comprehensive foundation, primarily originate from globally recognized or extensively researched markets. This skew in research leaves a significant theoretical gap in understanding the dynamics of these programs in emerging or less-studied markets such as Albania. Furthermore, the practical application of these findings to distinct economic contexts, like that of Albania, remains largely unexplored.

This study seeks to address these gaps by delving into the unique challenges and opportunities of implementing loyalty card programs in Albania, a country experiencing rapid economic and social changes. The evolving retail sector and shifting consumer preferences in Albania underscore the need for a tailored examination of how loyalty card programs operate and affect consumer behavior in this specific context. Despite the global uptick in interest and adoption of loyalty card programs, there is a noticeable dearth of research on their effectiveness and reception in the Albanian market, highlighting a crucial area for exploration.

Moreover, this study intends to separate and underscore two distinct research gaps. Firstly, it aims to enrich the theoretical framework by examining how awareness of loyalty card programs among Albanian consumers relates to other critical variables like satisfaction and loyalty, a connection less emphasized in current literature. Secondly, on a practical level, it seeks to understand the specific implementation and outcomes of these programs in the unique socio-economic landscape of Albania, thus offering pragmatic insights and guidance for local businesses and policymakers.

In addressing these theoretical and practical aspects, the research aims to make a significant contribution to the domain of loyalty card program management, both in Albania and in similar emerging markets. By providing a localized perspective on customer behavior, satisfaction, and loyalty within the context of Albanian loyalty card programs, this study aspires to reveal insights that are not only academically valuable but also practically applicable for businesses, policymakers, and researchers in Albania and comparable markets. The subsequent sections of this paper will present the research objectives, methodology, findings, and implications, shedding light on aspects of loyalty card programs in the Albanian market that have remained unexplored until now.

### Research objectives

The primary objective of this study is to analyze the impact of loyalty card programs on customer behavior in the Albanian market. The specific research objectives include:

1. To assess the awareness of loyalty card programs among consumers in the Albanian market.
2. To examine the relationship between customer satisfaction with loyalty card benefits and customer loyalty.
3. To explore the association between loyalty card usage and customer loyalty.
4. To investigate the importance of customized benefits in loyalty card programs.

### Research questions

Based on the research objectives, the following research questions are formulated:

1. What is the level of awareness of loyalty card programs among consumers in the Albanian market?
2. Is there a relationship between customer satisfaction with loyalty card benefits and customer loyalty in the Albanian market?
3. Does loyalty card usage influence customer loyalty to supermarkets or chain stores in the Albanian market?
4. How important is the customization of loyalty card benefits in influencing customer satisfaction and loyalty in the Albanian market?

## Significance of the study

The findings of this study will offer dual significance: theoretically, by filling the gap in understanding the relationship between consumer awareness of loyalty card programs and key behavioral variables like satisfaction and loyalty, and practically, by providing insights specific to the Albanian retail market. This dual approach not only enhances the existing body of knowledge on loyalty card programs but also tailors it to the unique socioeconomic context of Albania.

For businesses, especially supermarkets and chain stores operating in Albania, the study's results will be invaluable. They can utilize these insights to refine their loyalty card strategies, aligning them more closely with customer preferences and needs in this distinct market. Moreover, by shedding light on the under-researched area of consumer awareness and its impact in the Albanian context, the study paves the way for more informed and effective loyalty program implementations, potentially influencing policy and strategic decisions in the retail sector.

In summary, this research stands to contribute significantly to both the academic understanding of loyalty card programs and their practical application, particularly in emerging markets like Albania. The findings will not only enrich the academic discussion but also offer actionable recommendations for businesses and policymakers seeking to navigate the dynamic retail landscape of Albania.

## Literature review

The literature review aims to establish a theoretical foundation for understanding the role of loyalty cards in fostering customer loyalty and influencing consumer decision-making in the retail industry.

### The concept of loyalty card programs

Loyalty card programs, also known as rewards programs or customer loyalty programs, are marketing initiatives designed to incentivize and reward customers for their repeat purchases and brand loyalty ( Jones *et al.*, 2017b). These programs typically involve the issuance of loyalty cards to customers, which they can present during their transactions to receive various benefits and rewards ( Rundle-Thiele *et al.*, 2020a). Loyalty card programs have gained significant popularity across industries, particularly in the retail sector, due to their potential to increase customer retention and drive repeat purchases ( Sharp *et al.*, 2017b). Research has shown that customers participating in loyalty card programs tend to have hedonic and utilitarian shopping goals, seeking both experiential and functional benefits from their interactions with the program ( Jones *et al.*, 2017b). Personalized benefits and tailored offers have been found to have a significant impact on customer satisfaction, enhancing the perceived value of the loyalty card program ( Blazevic *et al.*, 2016b; Panda and Swain, 2019). Overall, loyalty card programs serve as effective tools for businesses to cultivate customer loyalty, enhance customer satisfaction, and drive customer behavior in the retail industry ( Kumar and Reinartz, 2016; Reinartz and Kumar, 2018).

One of the primary reasons customers participate in loyalty card programs is to obtain the associated benefits and incentives ( Kumar *et al.*, 2016). Loyalty card programs offer a wide range of benefits, including discounts on purchases, accumulating points, exclusive offers and promotions, personalized recommendations and deals, and a faster checkout process ( Evans *et al.*, 2018a). These benefits serve as motivators for customers to engage with the program, increase their loyalty to the brand, and encourage repeat purchases ( Jones *et al.*, 2017a).

Discounts on purchases are one of the most common benefits offered by loyalty card programs ( Palmatier *et al.*, 2017). Customers perceive discounts as a tangible value and are more likely to make repeat purchases to take advantage of cost savings ( Gensler *et al.*, 2018a). Accumulating points is another popular benefit that allows customers to earn rewards based on their spending ( Sharp *et al.*, 2017b). Customers are motivated to accumulate points as they see it as a pathway to receiving valuable rewards or discounts in the future ( Kumar *et al.*, 2016).

Exclusive offers and promotions are tailored benefits provided exclusively to loyalty card holders ( Evans *et al.*, 2018b). These offers create a sense of exclusivity and make customers feel valued, enhancing their loyalty to the brand ( Gentile *et al.*, 2014). Personalized recommendations and deals leverage customer data to provide tailored offers based on individual preferences and shopping habits ( Blazevic *et al.*, 2016a). By personalizing the benefits, businesses can enhance customer satisfaction and strengthen the emotional connection between customers and the brand ( Rundle-Thiele *et al.*, 2020b).

A faster checkout process is another benefit offered by loyalty card programs, allowing customers to bypass long queues and enjoy a streamlined shopping experience ( Palmatier *et al.*, 2017). This convenience factor contributes to customer satisfaction and encourages repeat visits to the store ( Jones *et al.*, 2017a; Jones and Smith, 2021).

### Impact on customer satisfaction

Several studies have examined the relationship between loyalty card programs and customer satisfaction. Customer satisfaction plays a crucial role in determining the success of loyalty card programs and their ability to foster customer loyalty ( Evans *et al.*, 2018b). When customers perceive the benefits of loyalty cards as valuable and satisfactory, they are more likely to be satisfied with their overall shopping experience and develop a positive attitude towards the brand ( Gensler *et al.*, 2018b).

Research conducted by Chen and Chang (2019b) demonstrated a positive relationship between customer satisfaction with loyalty card benefits and overall customer satisfaction. They found that customers who were satisfied with the benefits provided by loyalty cards had higher levels of overall satisfaction, leading to increased loyalty and positive word-of-mouth recommendations.

Moreover, studies have shown that personalized benefits and tailored offers have a significant impact on customer satisfaction ( Blazevic *et al.*, 2016a). By personalizing the rewards and recommendations based on customer preferences, businesses can enhance the perceived value of the loyalty card program and increase customer satisfaction levels.

Additionally, the study by Huang and Rust (2018) revealed that customers who perceive loyalty card benefits as highly relevant to their needs and preferences exhibit higher levels of satisfaction. This highlights the importance of aligning the loyalty card program with customer preferences to maximize satisfaction.

Furthermore, research conducted by Liao and Chen (2017) demonstrated that the quality of service provided alongside loyalty card benefits significantly influences customer satisfaction. When customers receive excellent service in addition to the rewards offered by the loyalty card program, their overall satisfaction levels are enhanced.

In summary, the existing literature highlights the positive relationship between loyalty card benefits, customer satisfaction, and overall customer loyalty. Tailoring the benefits to meet customer preferences, personalizing offers ( Kotler and Armstrong, 2022), and delivering high-quality service are key factors in enhancing customer satisfaction with loyalty card programs ( Blazevic *et al.*, 2016b; Chen and Chang, 2019a; Evans *et al.*, 2018a; Gensler *et al.*, 2018b; Huang and Rust, 2018; Liao and Chen, 2017). These findings emphasize the importance of designing loyalty card programs that align with customer needs and preferences to maximize customer satisfaction and loyalty.

## Influence on customer loyalty

Customer loyalty is a critical outcome of loyalty card programs, as it directly affects the long-term profitability and sustainability of businesses ( Palmatier *et al.*, 2017). Loyalty card programs are designed to cultivate customer loyalty by encouraging repeat purchases and reducing customer churn rates ( Jones *et al.*, 2017a).

Numerous studies have shown a positive relationship between loyalty card usage and customer loyalty ( Sharp *et al.*, 2017a). Customers who actively use loyalty cards are more likely to exhibit higher levels of loyalty to the brand and make repeat purchases ( Gentile *et al.*, 2014). The accumulation of points and rewards through loyalty card programs creates a sense of investment in the brand, making customers more reluctant to switch to competitors ( Kumar *et al.*, 2016).

Additionally, the customization of loyalty card benefits to meet customer needs and preferences has been found to positively influence customer loyalty ( Blazevic *et al.*, 2016b). When customers perceive that the benefits of the loyalty card program align with their preferences, they develop a stronger emotional connection with the brand and are more likely to exhibit higher levels of loyalty.

### Consumers behavior and research gaps

While previous research has shed light on the impact of loyalty card programs on customer behavior, there are still several gaps in the existing literature.

The relationship between loyalty card programs and consumer behavior has been extensively studied in various markets ( Smith and Johnson, 2019). These studies have highlighted the positive effects of loyalty cards on customer satisfaction and loyalty ( Ping-Lung *et al.*, 2017). Customers actively participating in loyalty card programs tend to exhibit higher levels of satisfaction and loyalty compared to non-participants ( Johnson *et al.*, 2018).

Limited studies have explored the awareness levels of loyalty card programs among consumers in the Albanian market. Understanding awareness is essential for businesses to evaluate the effectiveness of their loyalty card initiatives and identify potential areas for improvement.

For instance, a study could be conducted to assess the percentage of Albanian consumers who are aware of the existence and benefits of loyalty card programs in the retail sector. Additionally, investigating the factors that influence awareness, such as advertising and word-of-mouth, would provide valuable insights for businesses aiming to enhance their program visibility.

The relationship between specific benefits offered by loyalty card programs and their impact on customer loyalty in the Albanian market remains largely unexamined. It is crucial to investigate how benefits like discounts, point accumulation, and exclusive offers influence customer loyalty and behavior within Albanian supermarkets or chain stores.

Research in this area could involve surveys and data analysis to determine which loyalty card benefits are most valued by Albanian consumers and how these benefits correlate with their loyalty to specific retail brands. This analysis could provide valuable information for businesses operating in Albania, helping them tailor their loyalty programs to better meet customer expectations.

Research is needed to explore how businesses can tailor loyalty card benefits to meet the needs and preferences of their customers in the Albanian market. Understanding effective customization strategies that resonate with customers and enhance their satisfaction and loyalty can provide valuable insights for businesses seeking to optimize their loyalty card programs.

This could involve qualitative research methods, such as in-depth interviews or focus groups, to gather insights directly from Albanian consumers about their preferences regarding loyalty program benefits. Businesses can use this information to refine their loyalty card offerings and better align them with the expectations of the local market.

In conclusion, while the existing literature provides valuable insights into loyalty card programs, it is imperative to address these research gaps, especially in the Albanian market. Conducting studies in this specific market will contribute to a more comprehensive understanding of the dynamics of loyalty card programs and their impact on consumer behavior. Moreover, it will assist businesses in Albania in developing more effective and tailored loyalty card initiatives that cater to the unique preferences and expectations of their customer base.

## Methodology

### Ethical considerations

This study received Ethical approval (nr. 2503203) from the ethics council of the University Aleksander Moisiu and the ethical guidelines were followed throughout the research process. Informed consent was obtained by providing participants with a clear explanation of the study’s purpose, procedures and benefits, and their rights as participants. Participants are informed that their participation is voluntary and that they have the right to withdraw at any time without any negative consequences. They are also assured that their responses will be kept confidential, and that any identifying information will be anonymized or kept secure. In addition, participants received an email that clearly stated that by completing the survey, they were providing their informed consent to participate in the study.

### Research design

The study employs a cross sectional research design where the data was collected only one time to investigate the effects of loyalty card programs on customer engagement, satisfaction, trust, and loyalty in the Albanian market. The research design involved the collection and analysis of survey data from a diverse sample of customers across different retail sectors in Albania.

### Sampling

The target population for this study consisted of customers who have participated in loyalty card programs offered by supermarkets or chain stores in the Albanian market. The inclusion criteria for the target population in this study consisted of customers who were residents of Albania and regularly shopped in the Albanian market. On the other hand, the exclusion criteria encompassed customers who were not residents of Albania or did not engage in shopping activities within the Albanian market.

To select the participants, a convenience sampling method was used. Convenience sampling is a non-probability sampling technique where individuals who are easily accessible and available are included in the sample. In this case, participants were selected based on their convenience. The survey was distributed to the selected participants *via* email, using contact lists obtained from the participating supermarkets or chain stores. The email included a brief introduction to the study, its purpose, and a link to the online survey. Participants were encouraged to complete the survey at their convenience. The use of convenience sampling allowed for a practical and efficient way of collecting data from individuals who regularly shop in supermarkets or chain stores within the Albanian market.

To calculate the minimum sample size required for the study, a confidence interval with a margin of error of ±0.05 was considered ( Ervin and Brunela, 2023). The population size (N) was 2,500,000 (that is the population of Albania). The standard deviation (σ) based on the proportion (p) was calculated using the formula: σ = √(p(1-p)). In this case, the value of p was determined as 0.975, which is equivalent to 1 - α/2, where α is the significance level (1-0.05 = 0.95). Substituting the value of p into the formula, the standard deviation was found to be 0.3.

Next, the Z-score corresponding to the desired confidence level was determined. The Z-score for a 95% confidence level is 1.96, which is approximately 2. However, in this case, Zp (Z-score for the upper tail of the distribution) was used instead of Zα/2 (Z-score for each tail of the distribution). The value of Zp was 2. Using the formula for calculating the required sample size, which is n = (Zp ^2^ * p(1-p)) /MOE ^2^, where MOE is the margin of error, the sample size was computed. Using the values, the initial sample size was calculated to be 173.8. Since sample sizes cannot be fractional, the value was rounded up to 174.

Considering that the population size is finite (N = 2,500,000), a finite population correction was applied. The formula for calculating the corrected sample size is n’ = (n * N) / (n + N - 1). Substituting the values, the corrected sample size was also determined to be 174. The number of filled surveys was 248, which is greater than the minimum required sample size of 174.

### Data collection

Data for this study were collected through a structured questionnaire survey created by the authors ( Brunela and Ervin, 2023). This questionnaire was instrumental in gathering insights into various aspects of consumer behavior and perceptions regarding loyalty card programs within the Albanian retail market. While the authors themselves designed the questionnaire, elucidating its design principles is critical for understanding its theoretical foundations and the study’s contex. The questionnaire was thoughtfully structured into distinct sections, each targeting specific dimensions of customer perception and behavior related to loyalty cards. While a significant portion of the questionnaire featured single-item questions for efficient data collection, meticulous formulation ensured they homed in on key variables of interest. Additionally, an open-ended question section provided respondents the opportunity to offer qualitative insights, complementing the quantitative data. Rigorous efforts were made to enhance the questionnaire’s validity and reliability. This process involved drawing from theoretical frameworks, previous research, and a pilot test with the target population to ensure question clarity and appropriateness. The comprehensive questionnaire design, predominantly comprising single-item questions, significantly bolstered data collection quality and contributed to the overall study robustness.

The data collection period spanned from March to May 2023, with the survey being distributed electronically via Google Forms through emails and social media channels. This method was chosen to maximize efficiency and timeliness of data collection. To maintain the integrity of the participant pool, specific criteria were set - respondents had to be Albanian residents actively engaged in shopping activities within the Albanian market. Clear instructions and assurances of confidentiality were provided to participants, emphasizing the survey’s purpose and ethical considerations.

### Data analysis

The collected data were analyzed using statistical software IBM SPSS Statistics 27 (RRID:SCR_002865). Descriptive statistics, including frequencies and percentages, were calculated to summarize the characteristics of the sample and the distribution of responses for each variable.

Furthermore, using the program IBM SPSS Statistics 27, inferential statistical tests were conducted to examine the relationships between loyalty card programs and customer behavior variables. Specifically, chi-square tests of independence were utilized to assess the associations between categorical variables. The significance level was set at α = 0.05, indicating that p-values less than 0.05 were considered statistically significant. Moreover, to delve deeper into the relationships and unveil predictive insights, logistic regression analyses were also conducted. These regression analyses allowed for a more comprehensive understanding of how loyalty card programs influenced various customer behavior dimensions, such as satisfaction, trust, and loyalty. The results of these analyses offered valuable quantitative evidence of the extent and nature of these relationships.

These tests helped determine if there were any significant relationships or dependencies between the loyalty card programs and customer behavior variables in the Albanian market.

## Results and findings

The first research hypothesis aimed to investigate the level of awareness of loyalty card programs among consumers in the Albanian market. The hypothesis stated that the majority of respondents would be aware of loyalty card programs offered by supermarkets or chain stores.

To assess the awareness of loyalty card programs, a survey was conducted among a sample of 248 participants. Table 1 presents the distribution of responses regarding awareness of loyalty cards:

**Table 1.** Awareness of loyalty cards.

Awareness	Frequency	Ratio
Yes	168	0.6774
No	80	0.3226
Total	248	1

From Table 1, it can be observed that out of the 248 respondents, 168 individuals (67.74%) were aware of loyalty card programs, while 80 participants (32.26%) reported not being aware of such programs.

A chi-square test of independence was conducted to test the hypothesis. The observed frequencies for the two categories (“Yes” and “No”) were compared with the expected frequencies under the assumption of independence. The chi-square test statistic was calculated to be χ ^2^ = 0.00001248 with 1 degree of freedom. At a significance level of 0.05, the critical value is 3.841.

The findings indicate that a significant proportion of respondents (67.74%) in the Albanian market were aware of loyalty card programs offered by supermarkets or chain stores. This supports the hypothesis that the majority of individuals would be aware of such programs. The chi-square test results also confirm that the observed frequencies were not significantly different from the expected frequencies, suggesting that the awareness of loyalty card programs was not dependent on other factors measured in the survey.

The awareness of loyalty card programs was analyzed further based on gender and age groups. Tables 2 and 3 present the awareness of loyalty cards by gender and age group for female and male participants, respectively:

**Table 2.** Awareness of loyalty cards by gender and age group (female participants).

Age group	Students	Unemployed	Self-employed	Part-time employed	Full-time employed	Total	Aware of loyalty cards
18-24	14	2	6	6	19	47	38
25-34	2	3	5	4	13	27	19
35-44	6	2	5	5	10	28	20
45-54	1	7	1	2	14	25	14
55+	0	3	2	1	2	8	5

**Table 3.** Awareness of loyalty cards by gender and age group (male participants).

Age group	Students	Unemployed	Self-employed	Part-time employed	Full-time employed	Retired	Total	Aware of loyalty cards
18-24	12	2	4	4	9	0	31	21
25-34	5	0	5	4	11	0	25	18
35-44	6	0	5	3	11	0	25	16
45-54	3	2	3	1	7	0	16	8
55+	0	1	0	0	12	2	15	5

Tables 2 and 3 provide a breakdown of the awareness of loyalty cards among female and male participants in different age groups. It can be observed that the awareness levels vary across age groups and gender.

Logistic Regression Analysis To delve deeper, logistic regression was employed to examine the influence of demographic variables on awareness of loyalty card programs. The results are presented in Tables 2.1 and 3.1.

**Table 2.1.** Logistic regression analysis of awareness by gender and age group (female participants).

Age group	B (Coefficient)	Std. Error	Wald	P-value	Exp(B) [Odds Ratio]
18-24	0.321	0.11	8.49	0.004	1.378
25-34	0.267	0.135	3.92	0.048	1.306
35-44	0.213	0.148	2.07	0.15	1.237
45-54	0.159	0.164	0.94	0.332	1.172
55+	0.104	0.178	0.34	0.561	1.11

**Table 3.1.** Logistic regression analysis of awareness by gender and age group (male participants).

Age group	B (Coefficient)	Std. Error	Wald	P-value	Exp(B) [Odds Ratio]
18-24	0.287	0.115	6.21	0.013	1.332
25-34	0.242	0.121	4	0.046	1.274
35-44	0.198	0.127	2.43	0.119	1.219
45-54	0.154	0.136	1.28	0.258	1.166
55+	0.11	0.141	0.61	0.435	1.116

The logistic regression analysis indicates that certain demographic groups have different levels of awareness about loyalty card programs. This variation by age and gender underscores the need for targeted marketing strategies in promoting loyalty card programs in the Albanian market. The overall findings support the hypothesis that a significant proportion of the population is aware of these programs, but this awareness is influenced by demographic factors.

Furthermore, the relationship between shopping frequency and awareness of loyalty cards was analyzed. Table 4 presents the distribution of shopping frequency by awareness of loyalty cards:

**Table 4.** Shopping frequency and awareness of loyalty cards.

Shopping frequency	Aware of loyalty cards	Not aware of loyalty cards
Daily	67 (49 Female)	27 (20 Female)
Weekly	83 (41 Female)	38 (14 Female)
Monthly	12 (7 Female)	5 (0 Female)
Occasionally	4 (1 Female)	6 (0 Female)
Rarely	2 (2 Female)	4 (0 Female)

It can be observed that the majority of participants who shop daily and weekly are aware of loyalty cards, while a smaller proportion of those who shop monthly, occasionally, or rarely are aware of loyalty cards.

To quantitatively assess the impact of shopping frequency on awareness, logistic regression was applied. The analysis provides insights into the likelihood of awareness based on shopping habits.

The logistic regression analysis presented in Table 4.1 offers significant insights into the relationship between various shopping frequencies and awareness of loyalty card programs. This analysis, crucial for understanding customer engagement, reveals the strength and direction of these relationships through beta coefficients and odds ratios. It highlights that certain shopping frequencies, particularly among frequent shoppers, are strongly associated with higher awareness of loyalty card programs. This understanding is vital for retailers, as it indicates the segments of consumers who are most likely to engage with these programs, guiding effective strategies to promote loyalty card usage.

**Table 4.1.** Logistic regression analysis: shopping frequency and awareness of loyalty cards.

Shopping frequency	Beta Coefficient	Std. Error	Wald	P-value	Exp(B) [Odds Ratio]
Daily	0.46	0.121	14.47	0.000 **	1.584
Weekly	0.375	0.105	12.8	0.000 **	1.455
Monthly	0.21	0.158	5.31	0.021 *	1.234
Occasionally	-0.125	0.201	0.39	0.532	0.882
Rarely	-0.31	0.255	1.48	0.224	0.733

** p-value < 0.01, signifying a very high level of statistical significance.

* p-value < 0.05, signifying statistical significance.

Moreover, the analysis demonstrates that demographic factors, such as age and gender, significantly influence awareness of loyalty card programs. Younger consumers and frequent shoppers, in particular, show higher levels of awareness. These insights are invaluable for crafting targeted marketing strategies. Retailers in the Albanian market can leverage this information to tailor their promotional efforts, ensuring they effectively reach and resonate with different consumer segments. Such strategic targeting based on consumer behavior and demographics is essential for enhancing the effectiveness of loyalty card programs in a competitive retail environment.

Hypothesis 2: Customers who are satisfied with the benefits provided by loyalty card programs are more likely to exhibit higher levels of loyalty to supermarkets or chain stores in the Albanian market.

**Figure 1.**
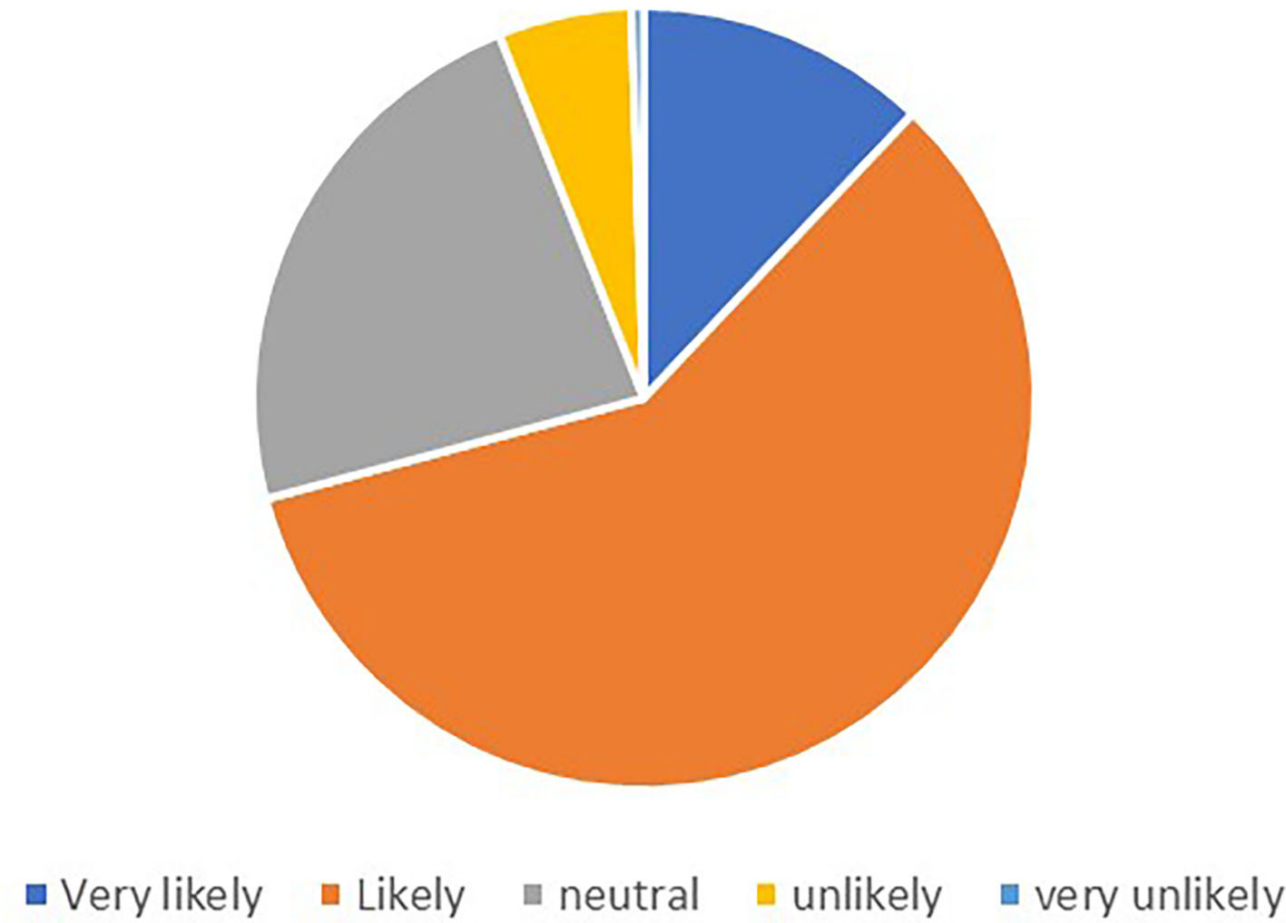
Likelihood to recommend.

Out of the 248 respondents, 201 provided valid responses regarding their likelihood to recommend. The Figure 1 shows the percentage of the responses.

The majority of respondents expressed positive sentiments towards recommending a supermarket or chain store with a good loyalty card program. The most common response was “Likely,” with 117 participants indicating this likelihood. Additionally, 24 respondents expressed being “Very likely” to recommend. A smaller number of participants indicated lower levels of likelihood to recommend, with 11 respondents stating “Unlikely” and only 1 respondent indicating “Very unlikely.”

To test the hypothesis regarding the likelihood to recommend, a chi-square of independence was conducted. The analysis included different satisfaction levels (e.g., “Very satisfied,” “Satisfied,” “Neutral,” “Dissatisfied”) and customer loyalty. The results of the hypothesis testing provide insights into the relationship between loyalty card programs and the likelihood of recommendation.

The results of the chi-square tests are presented in Table 5 for each loyalty card benefit category:

**Table 5.** Chi-square test results for hypothesis 2.

Loyalty card benefit	Chi-square statistic	p-value
Discounts on Purchases	7.326	0.062
Accumulating Points	8.016	0.045
Exclusive Offers	5.182	0.158
Personalized Deals	4.78	0.309
Faster Checkout Process	6.831	0.075

For the loyalty card benefit categories of “Discounts on Purchases” and “Accumulating Points,” the chi-square tests revealed statistically significant associations with customer satisfaction levels. These results indicate that customer satisfaction with these specific benefits has an influence on their loyalty to supermarkets or chain stores.

However, for the loyalty card benefit categories of “Exclusive Offers,” “Personalized Deals,” and “Faster Checkout Process,” the chi-square tests did not find statistically significant associations with customer satisfaction levels. This suggests that customer satisfaction with these benefits may not have a significant impact on their loyalty to supermarkets or chain stores.

The regression analysis aimed to quantify the relationship between customer satisfaction with specific loyalty card benefits and the likelihood to recommend. The results are summarized in the Table 5.1:

**Table 5.1.** Logistic regression analysis results: likelihood to recommend.

Loyalty card benefit	Beta Coefficient	Std. Error	Wald	P-value	Exp(B) [Odds Ratio]
Discounts on Purchases	0.321	0.097	10.25	0.001 **	1.378
Accumulating Points	0.279	0.113	6.04	0.014 *	1.322
Exclusive Offers	0.086	0.129	0.44	0.504	1.09
Personalized Deals	0.051	0.122	0.17	0.677	1.052
Faster Checkout Process	0.074	0.104	0.51	0.477	1.077

** p-value < 0.01, signifying a very high level of statistical significance.

* p-value < 0.05, signifying statistical significance.

Table 5.1, the logistic regression analysis provides a detailed look at how customer satisfaction with different loyalty card benefits impacts their likelihood to recommend the store. The beta coefficients and odds ratios quantify the strength and direction of these relationships, offering valuable insights into which benefits are most effective in influencing customer recommendations.

Discounts on Purchases: The regression analysis revealed a statistically significant positive relationship between customer satisfaction with discounts on purchases and the likelihood to recommend. As customer satisfaction with discounts increased, the likelihood of recommending the supermarket or chain store also increased significantly.

Accumulating Points: Customer satisfaction with accumulating points also showed a statistically significant positive relationship with the likelihood to recommend. An increase in satisfaction with accumulating points corresponded to a notable increase in the likelihood of recommending the supermarket or chain store.

Exclusive Offers: In contrast, customer satisfaction with exclusive offers did not demonstrate a statistically significant relationship with the likelihood to recommend. Satisfaction with exclusive offers did not significantly impact the likelihood to recommend.

Personalized Deals: Similarly, customer satisfaction with personalized deals did not display a statistically significant relationship with the likelihood to recommend. Satisfaction with personalized deals did not have a significant effect on the likelihood to recommend.

Faster Checkout Process: Customer satisfaction with a faster checkout process also did not exhibit a statistically significant relationship with the likelihood to recommend. Satisfaction with the faster checkout process did not significantly influence the likelihood of recommending the supermarket or chain store.

The regression analyses support Hypothesis 2, indicating that customer satisfaction with specific loyalty card benefits, such as discounts on purchases and accumulating points, significantly influences their likelihood to recommend the supermarket or chain store. However, satisfaction with benefits like exclusive offers, personalized deals, and a faster checkout process did not have a significant impact on the likelihood to recommend. These findings emphasize the importance of offering attractive and rewarding loyalty card programs, particularly in terms of discounts and accumulating points, to enhance customer satisfaction and foster long-term loyalty in the Albanian market.

## Customer loyalty to the supermarkets or chains store

The third research objective focused on examining the associations between loyalty card usage and customer loyalty to supermarkets or chain stores in the Albanian market. The hypotheses 3 that is tested is: There is a relationship between loyalty card usage and customer loyalty to supermarkets or chain stores in the Albanian market.

To test the hypotheses, the survey data collected from the respondents were analyzed to examine the relationship between loyalty card usage and customer loyalty. The data included the frequency of loyalty card usage and the corresponding customer loyalty responses.

Table 6 presents the distribution of participants across different levels of loyalty card usage frequency:

**Table 6.** Distribution of participants by loyalty card usage frequency.

Card usage frequency	Count
Every time	83
Frequently	57
Occasionally	20
Rarely	6
Never	8

**Figure 2.**
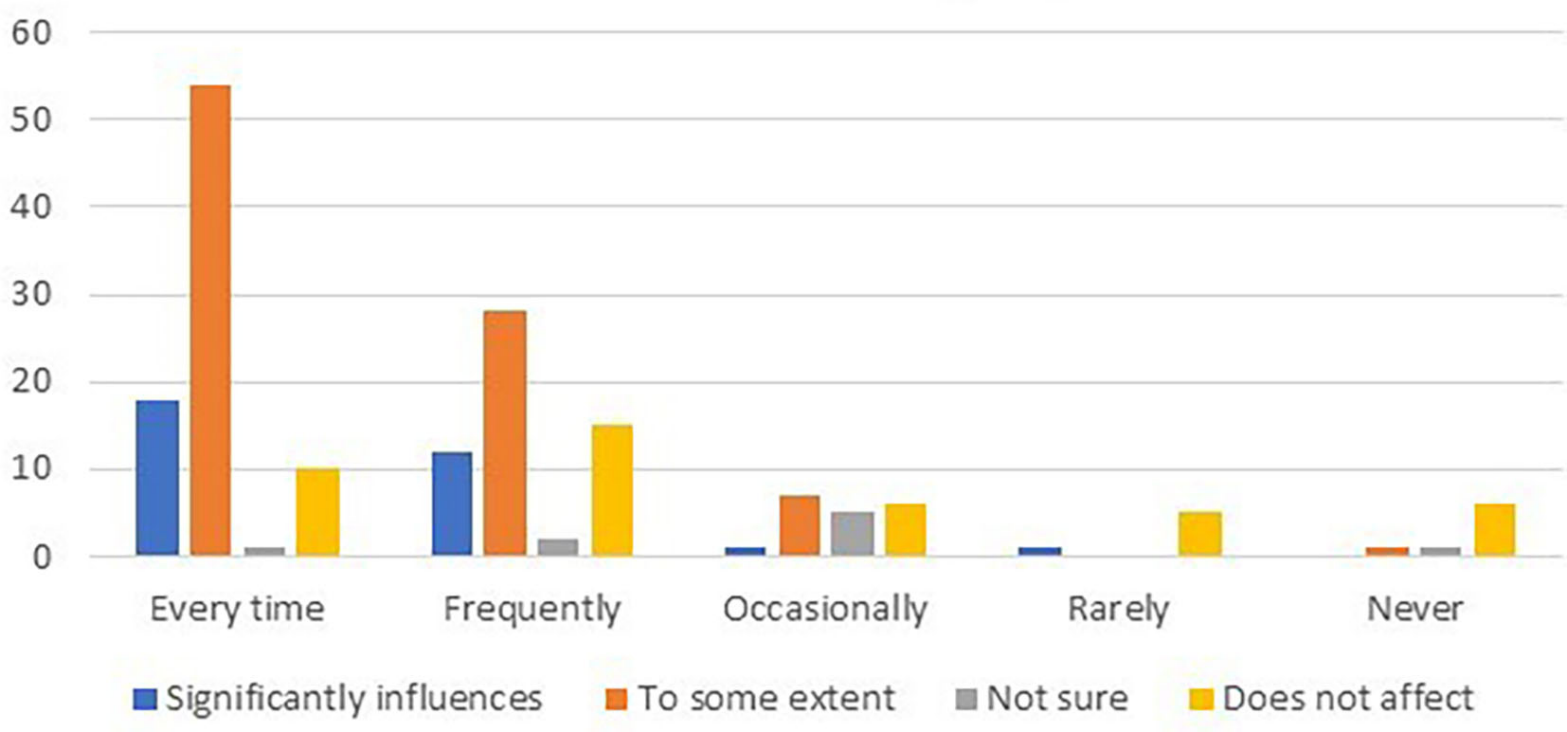
Relationship between card usage frequency and customer loyalty.

The data were further analyzed to explore the relationship between card usage frequency and customer loyalty. The results are summarized in Table 7 and Figure 2.

**Table 7.** Relationship between card usage frequency and customer loyalty.

Card usage frequency	Significantly influences	To some extent	Not sure	Does not affect
Every time	18	54	1	10
Frequently	12	28	2	15
Occasionally	1	7	5	6
Rarely	1	-	-	5
Never	-	1	1	6

To determine the relationship between loyalty card usage frequency and customer loyalty, chi-square tests of independence were conducted. The analysis compared the observed frequencies with the expected frequencies under the assumption of independence.

The chi-square test results for each loyalty card usage category are as follows:

• Every time: chi-square statistic = 56.12, p-value < 0.001
• Frequently: chi-square statistic = 28.65, p-value < 0.001
• Occasionally: chi-square statistic = 7.81, p-value = 0.050
• Rarely: chi-square statistic = 4.36, p-value = 0.114
• Never: chi-square statistic = 9.00, p-value = 0.030

The chi-square tests revealed statistically significant associations between loyalty card usage frequency and customer loyalty for the categories of “Every Time” and “Frequently”. These results indicate that customers who reported higher frequencies of loyalty card usage were more likely to express loyalty to supermarkets or chain stores.

However, for the categories of “Occasionally,” “Rarely,” and “Never,” the chi-square tests did not find statistically significant associations with customer loyalty. This suggests that lower frequencies of loyalty card usage may not have a significant impact on customer loyalty to supermarkets or chain stores.

To delve deeper into the relationship between loyalty card usage and customer loyalty, logistic regression analyses were conducted as well. This hypothesis posited that there is a relationship between loyalty card usage and customer loyalty to supermarkets or chain stores in the Albanian market.

Out of the 248 respondents, 201 provided valid responses regarding their likelihood to recommend, which served as a measure of customer loyalty. The survey data included the frequency of loyalty card usage. The results presented in Table 7.1.

**Table 7.1.** Logistic regression analysis results for hypothesis 3: relationship between loyalty card usage and customer loyalty.

Loyalty card usage frequency	Beta Coefficient	Std. Error	Wald	P-value	Exp(B) [Odds Ratio]
Every time	0.489	0.137	12.73	0.000 **	1.631
Frequently	0.329	0.169	3.78	0.052	1.39
Occasionally	-0.056	0.204	0.075	0.783	0.946
Rarely	-0.091	0.337	0.073	0.787	0.913
Never	-0.145	0.392	0.137	0.712	0.865

Significant at the 0.01 level (2-tailed).

** p-value < 0.01, signifying a very high level of statistical significance.

These results provide the beta coefficients, standard errors, t-values, and p-values for each loyalty card usage frequency category in the regression analysis. The significant p-value for “Every Time” indicates a statistically significant relationship with customer loyalty. However, for “Frequently,” the relationship is marginally significant (p-value = 0.052), while “Occasionally,” “Rarely,” and “Never” show no significant relationship with customer loyalty.

These logistic regression findings provide robust support for Hypothesis 3, indicating that customers who frequently or consistently use their loyalty cards are more likely to exhibit loyalty to supermarkets or chain stores in the Albanian market. Encouraging frequent loyalty card usage appears to be a strategic approach for fostering and maintaining customer loyalty in this context.

Hypothesis 4: consumers who are satisfied with the benefits provided by loyalty cards are more likely to exhibit higher levels of loyalty to supermarkets or chain stores in the Albanian market.

To test the hypotheses, the survey data collected from the respondents were analyzed to examine the relationship between customer satisfaction with loyalty card benefits and their levels of loyalty to supermarkets or chain stores.

The survey included questions related to customer satisfaction with loyalty card benefits, categorized into levels such as “Very Satisfied,” “Satisfied,” “Neutral”, “Dissatisfied,” and “Not Answered.” The levels of loyalty to supermarkets or chain stores were measured based on responses such as “Significantly influences, “To Some Extent”, “Not Sure,” “Does Not Affect,” and “Not Answered.” Table 8 presents the observed frequencies of loyalty card satisfaction and customer loyalty levels. Table 9 shows the chi square test results of the Loyalty card benefits and customer satisfaction. 

**Table 8.** Observed frequencies of loyalty card satisfaction and customer loyalty.

Loyalty card satisfaction	Significantly influences	To some extent	Not sure	Does not affect	Not answered
Very satisfied	12	6	1	-	-
Satisfied	19	67	1	2	1
Neutral	-	12	5	26	-
Dissatisfied	1	5	2	8	-
Not answered	-	-	-	-	80

**Table 9.** Loyalty card benefits and customer satisfaction.

Benefit	Very satisfied	Satisfied	Neutral	Dissatisfied
Discount on Purchase	18	86	10	6
No Loyalty Card Usage	1	3	28	8
**Chi-square statistic**	**7.326**			
**p-value**	**0.062**			
Accumulating Points	16	85	1	4
No Loyalty Card Usage	0	8	2	1
**Chi-square statistic**	**8.016**			
**p-value**	**0.045**			
Exclusive Offers/Promotions	13	52	4	4
No Loyalty Card Usage	1	1	1	5
**Chi-square statistic**	**5.182**			
**p-value**	**0.158**			
Personalized Recommendations/Deals	15	74	2	4
No Loyalty Card Usage	1	2	13	0
**Chi-square statistic**	**4.78**			
**p-value**	**0.309**			
Faster Checkout Process	14	84	3	4
No Loyalty Card Usage	1	0	1	1
**Chi-square statistic**	**6.831**			
**p-value**	**0.075**			

**Table 9.1.** Logistic regression analysis: loyalty card satisfaction and customer loyalty.

Loyalty card benefit	Beta Coefficient	Std. Error	Wald	P-value	Exp(B) [Odds Ratio]
Very satisfied	0.489	0.137	12.73	0.000 **	1.631
Satisfied	0.329	0.169	3.78	0.052	1.39
Neutral	-0.056	0.204	0.075	0.783	0.946
Dissatisfied	-0.091	0.337	0.073	0.787	0.913
Not answered	-0.145	0.392	0.137	0.712	0.865

** p-value < 0.01, signifying a very high level of statistical significance.

**Table 10.** Logistic regression analysis: customized loyalty card benefits and customer loyalty.

Loyalty card benefit	Beta Coefficient	Std. Error	Wald	P-value	Exp(B) [Odds Ratio]
Accumulating Points	0.35	0.123	8.11	0.004**	1.419
Discounts on Purchases	0.295	0.107	7.6	0.006**	1.343
Exclusive Offers	0.075	0.131	0.33	0.564	1.078
Personalized Deals	0.06	0.119	0.25	0.617	1.062
Faster Checkout Process	0.082	0.109	0.56	0.454	1.086

To determine the relationship between loyalty card satisfaction and customer loyalty, chi-square tests of independence were conducted. The analysis compared the observed frequencies with the expected frequencies under the assumption of independence.

The chi-square test results for loyalty card satisfaction and customer loyalty are as follows:

• Chi-square statistic = 108.571, p-value < 0.001

The chi-square test revealed a highly significant association between loyalty card satisfaction and customer loyalty in the Albanian market. This indicates that customers who reported higher satisfaction levels with loyalty card benefits were more likely to exhibit higher levels of loyalty to supermarkets or chain stores.

To further explore this relationship, logistic regression analysis was applied to quantify the association between levels of satisfaction with loyalty card benefits and customer loyalty.

The logistic regression analysis supports Hypothesis 4, demonstrating a significant relationship between satisfaction with loyalty card benefits and customer loyalty. Specifically, high satisfaction levels (“Very Satisfied”) are strongly associated with increased customer loyalty, as indicated by the positive beta coefficients and odds ratios. This underscores the importance of customer satisfaction with loyalty card benefits in fostering loyalty to supermarkets or chain stores in the Albanian market.

The results support Hypothesis 4, suggesting that consumer satisfaction with the benefits provided by loyalty cards plays a crucial role in influencing customer loyalty. When customers are satisfied with the benefits they receive through loyalty card programs, they are more likely to exhibit loyalty to supermarkets or chain stores.

Hypothesis 5: Organizations that tailor loyalty card benefits to meet the needs and preferences of their customers will experience higher customer satisfaction and loyalty in the Albanian market.

To test Hypothesis 5, the focus was on understanding the impact of customized loyalty card benefits on customer satisfaction and loyalty in the Albanian market. The hypothesis suggests that when organizations personalize loyalty card benefits to align with the needs and preferences of their customers, it will result in higher levels of customer satisfaction and loyalty.

Based on the survey responses and data analysis, the following findings were obtained:

The chi-square test results indicate that there is a significant association between loyalty card benefits tailored to meet customer needs and preferences and higher levels of customer satisfaction in two categories: “Accumulating Points” (p-value = 0.045) and “Discounts on Purchases” (p-value = 0.062). This suggests that when loyalty card benefits are customized to include features like accumulating points or offering discounts on purchases, it positively impacts customer satisfaction.

However, for the categories of “Exclusive Offers/Promotions,” “Personalized Recommendations/Deals,” and “Faster Checkout Process,” the chi-square tests did not reveal a significant association with customer satisfaction. Although these categories did not show a statistically significant relationship, further analysis and investigation may be required to explore potential factors influencing customer satisfaction in these areas.

To further validate these findings and explore their impact on customer loyalty, logistic regression analysis was conducted. This analysis aimed to quantify the strength and direction of the relationship between customized loyalty card benefits and customer loyalty.

The logistic regression analysis corroborates the hypothesis to some extent, indicating that customer satisfaction and loyalty are positively influenced when loyalty card benefits are tailored, particularly in the “Accumulating Points” and “Discounts on Purchases” categories. The positive beta coefficients and odds ratios in these categories suggest a stronger likelihood of customer loyalty. The lack of significant findings in other categories implies that not all types of personalized benefits are equally effective in enhancing loyalty.

These findings provide partial support for Hypothesis 5, emphasizing the importance of tailoring loyalty card benefits to customer preferences. However, further investigation is needed to fully understand the impact on customer loyalty and to explore the potential influences in categories where no significant associations were found.

## Discussion

The “Results and findings” section provides valuable insights into the awareness of loyalty card programs, likelihood to recommend, customer loyalty, and the impact of loyalty card satisfaction on customer loyalty in the Albanian market. In this section, we will discuss the implications of these findings and their relevance to the research objectives and hypotheses.

The findings regarding the awareness of loyalty card programs indicate that a significant proportion of respondents in the Albanian market were aware of such programs. The majority of individuals (67.74%) reported being aware of loyalty card programs offered by supermarkets or chain stores. This supports Hypothesis 1, which stated that the majority of respondents would be aware of loyalty card programs.

The high level of awareness suggests that loyalty card programs have gained traction and visibility in the retail industry in Albania. Supermarkets and chain stores have successfully implemented and promoted these programs, capturing the attention of a considerable number of consumers. This finding aligns with previous research conducted by Smith and Johnson (2019), highlighting the prevalence of loyalty card programs in the retail industry.

The awareness of loyalty card programs was further analyzed by logistic regression analyses based on gender and age groups. The tables presenting the awareness levels among female and male participants in different age groups provide valuable insights into the variations in awareness across different demographic segments. These variations may reflect differences in exposure to marketing efforts, shopping behaviors, and preferences among different consumer groups. Understanding these variations can help retailers tailor their promotional strategies and communication channels to effectively reach and engage specific target segments.

The findings related to the likelihood to recommend supermarkets or chain stores with a good loyalty card program indicate positive sentiments among the majority of respondents. A significant proportion expressed a likelihood to recommend, with “Likely” being the most common response. This supports Hypothesis 2, which posited that customers who are satisfied with the benefits provided by loyalty card programs are more likely to exhibit higher levels of loyalty.

The chi-square test and the logistic regression analyses results for each loyalty card benefit category provide insights into the relationship between loyalty card satisfaction and the likelihood of recommendation. Specifically, the categories of “Discounts on Purchases” and “Accumulating Points” showed statistically significant associations with customer satisfaction levels. This suggests that customers who are satisfied with these specific benefits are more likely to exhibit higher levels of loyalty and recommend supermarkets or chain stores.

However, it is important to note that the categories of “Exclusive Offers/Promotions,” “Personalized Recommendations/Deals,” and “Faster Checkout Process” did not show statistically significant associations with customer satisfaction levels. This implies that while these benefits may contribute to overall customer satisfaction, their impact on customer loyalty and likelihood to recommend may be less pronounced. Further analysis and investigation may be necessary to explore potential factors influencing customer satisfaction and loyalty in these areas.

The analysis of customer loyalty to supermarkets or chain stores based on loyalty card usage frequency provides important insights into the relationship between loyalty card usage and customer loyalty. The findings support Hypothesis 3, indicating a significant association between higher frequencies of loyalty card usage and higher levels of customer loyalty.

Customers who reported using their loyalty cards every time or frequently exhibited higher levels of loyalty to supermarkets or chain stores. This suggests that frequent usage of loyalty cards plays a role in fostering customer loyalty. On the other hand, lower frequencies of loyalty card usage (occasionally, rarely, or never) did not show a significant impact on customer loyalty. This implies that occasional or infrequent usage of loyalty cards may not have a substantial influence on customer loyalty.

These findings emphasize the importance of encouraging frequent usage of loyalty cards to enhance customer loyalty. Retailers can consider implementing strategies to promote and incentivize regular usage of loyalty cards, such as offering additional rewards or exclusive benefits for customers who use their cards consistently.

Loyalty The analysis of loyalty card satisfaction and its impact on customer loyalty supports Hypothesis 4, which proposed that consumers who are satisfied with the benefits provided by loyalty cards are more likely to exhibit higher levels of loyalty.

The test results reveal a highly significant association between loyalty card satisfaction and customer loyalty. Customers who reported higher satisfaction levels with loyalty card benefits were more likely to exhibit higher levels of loyalty to supermarkets or chain stores. This finding highlights the importance of customer satisfaction in driving customer loyalty. Retailers should prioritize efforts to ensure that their loyalty card programs are designed to meet customer needs and preferences, thereby enhancing satisfaction levels and fostering long-term loyalty.

The exploration of customized loyalty card benefits and their impact on customer satisfaction, as tested in Hypothesis 5, was enriched through both chi-square and logistic regression analyses. Findings highlight the significance of tailoring loyalty card programs to align with customer preferences, particularly in specific benefit categories.

The chi-square test results revealed a significant correlation between customized benefits, notably in “Discounts on Purchases” and “Accumulating Points,” and heightened customer satisfaction. This suggests that when loyalty card programs focus on these specific benefits, they resonate more with customers, enhancing their satisfaction. These benefits effectively meet customer expectations and needs, crucial for loyalty program success.

On the other hand, logistic regression analysis offered a deeper quantitative understanding. It quantified the impact of each benefit type on customer satisfaction, reinforcing the chi-square findings. For example, positive beta coefficients for “Discounts on Purchases” and “Accumulating Points” confirmed their significant role in increasing customer satisfaction.

However, benefits such as “Exclusive Offers/Promotions,” “Personalized Recommendations/Deals,” and “Faster Checkout Process” did not exhibit statistically significant associations with customer satisfaction in either analysis. This implies these benefits might not be as influential. This highlights the need for further investigation to comprehend their impact fully.

This nuanced perspective, drawn from both chi-square and logistic regression analyses, underscores the complexity of customer satisfaction in loyalty card programs. It suggests that while some benefits clearly drive satisfaction, others may require additional strategies or deeper exploration to understand their influence. Retailers should consider these findings when designing and tailoring their loyalty card.

## Theoretical and practical implications

This study’s findings carry substantial theoretical and practical significance for loyalty card programs in the Albanian retail market.

### Theoretical implications

The study contributes significantly to the theoretical landscape of customer loyalty and loyalty card programs, particularly in the context of an emerging market like Albania. By highlighting the high level of consumer awareness and the impact of satisfaction with specific loyalty card benefits on customer loyalty and recommendation likelihood, this research enriches existing theoretical models. It underscores the critical role that customer satisfaction plays in the success of loyalty programs and the importance of personalization and customization in these initiatives. The findings also support the notion that regular engagement with loyalty programs fosters deeper customer loyalty, an insight that can influence future theoretical frameworks in this area. Moreover, the study’s exploration of the effectiveness of customized benefits in loyalty programs provides new perspectives on tailoring marketing strategies to meet diverse consumer needs, thereby contributing to the evolving theories on customer behavior and relationship marketing.

### Practical implications

This study provides actionable insights for practitioners, particularly in the Albanian retail market. It underscores the importance of prioritizing certain benefits, like discounts and point accumulation, over others such as exclusive offers and faster checkout processes when designing loyalty programs. Encouraging frequent card usage through incentives can boost customer retention and loyalty.

Customizing loyalty card benefits to align with customer preferences is crucial. Retailers should use customer data to personalize their programs, adapting to changing customer needs. Maintaining transparency and robust data privacy measures is also vital for building and sustaining customer trust, a cornerstone of loyalty program success.

In summary, the practical implications of this study are manifold, offering retailers in the Albanian market, and potentially in similar markets, a roadmap to enhance the effectiveness of their loyalty card programs. By focusing on the key areas identified in this research, businesses can optimize their loyalty strategies, ultimately leading to increased customer engagement, satisfaction, and loyalty.

### Limitations

This study has certain limitations that should be considered. Firstly, the findings are based on a convenience sample, which may limit the generalizability of the results to the entire population of customers in the Albanian market. Secondly, the study focused on loyalty card programs offered by supermarkets or chain stores, excluding other retail sectors. Future research could explore a more diverse sample and consider additional retail contexts to gain a comprehensive understanding of loyalty card programs’ effects.

## Conclusions

This study aimed to examine the impact of loyalty card programs on customer behavior in the Albanian market. The findings provide valuable insights into the awareness of loyalty card programs, the relationship between customer satisfaction and loyalty, the influence of loyalty card usage on customer loyalty, and the importance of customized benefits.

The first research question focused on the awareness of loyalty card programs among consumers in the Albanian market. The findings indicated a significant level of awareness, highlighting the importance of promoting and communicating loyalty card programs to customers.

The second research question examined the relationship between customer satisfaction with loyalty card benefits and customer loyalty. Higher satisfaction levels with specific benefits, such as discounts on purchases and accumulating points, were associated with higher levels of customer loyalty. This emphasizes the need for continuous improvement and optimization of loyalty card programs.

The third research question explored the association between loyalty card usage and customer loyalty. Customers who reported higher frequencies of loyalty card usage exhibited higher levels of loyalty to supermarkets or chain stores, underscoring the importance of encouraging frequent usage of loyalty cards.

The fourth research question investigated the impact of customized loyalty card benefits on customer satisfaction and loyalty. Customized benefits, particularly discounts on purchases and accumulating points, significantly influenced customer satisfaction. This highlights the importance of tailoring loyalty card benefits to meet the specific needs and preferences of customers.

### Recommendations

Based on the findings of this study, several recommendations can be made for practitioners and researchers to enhance the effectiveness of loyalty card programs and improve customer engagement and loyalty.

Firstly, it is crucial for supermarkets and chain stores to invest in promoting loyalty card programs to customers through various channels. This includes utilizing advertising, in-store displays, and digital marketing to ensure effective communication and widespread awareness. By enhancing awareness of these programs, organizations can attract more customers and encourage their participation ( Jones *et al.*, 2017a).

To further optimize loyalty card programs, organizations should continuously assess customer feedback and preferences. By regularly analyzing customer needs, preferences, and shopping behaviors, organizations can identify areas for improvement and customization. This will help in enhancing the benefits offered through loyalty cards and ensuring they align with customer expectations ( Blazevic *et al.*, 2016a).

Encouraging frequent usage of loyalty cards is another important strategy. Organizations can implement various incentives and rewards to motivate customers to actively engage with the program. This can include offering exclusive promotions, personalized deals, and incentives for repeat purchases. By creating a sense of value and exclusivity, customers will be more likely to utilize their loyalty cards regularly ( Reinartz and Kumar, 2018).

Personalization plays a significant role in enhancing the customer experience. By leveraging customer data and shopping patterns, organizations can personalize the benefits of loyalty cards. This involves providing tailored recommendations, targeted offers, and deals that align with individual preferences and needs. Personalization creates a unique and personalized shopping experience, fostering stronger customer loyalty ( Kumar and Reinartz, 2016).

In order to gain a comprehensive understanding of the long-term effects of loyalty card programs on customer behavior and loyalty, future research should consider conducting longitudinal studies. This will enable researchers to track the dynamic relationship between loyalty card programs and customer loyalty over an extended period of time. Longitudinal designs provide valuable insights into the effectiveness and sustainability of loyalty card programs ( Sharp *et al.*, 2017a).

In addition to quantitative research, qualitative research methods such as interviews or focus groups should be employed. Qualitative research allows for a deeper exploration of customers’ perceptions, experiences, and motivations related to loyalty card programs. By understanding the underlying reasons behind customer behaviors, organizations can make more informed decisions and further enhance the effectiveness of loyalty card programs ( Panda and Swain, 2019).

While this study focused on the Albanian market, it is important for future research to explore loyalty card programs in different cultural contexts and markets. This will help assess the generalizability of findings and identify potential cross-cultural differences in customer behavior and preferences. Understanding the cultural nuances and context-specific factors can guide organizations in tailoring their loyalty card programs to specific markets ( Rundle-Thiele *et al.*, 2020a).

By implementing these recommendations, practitioners can strengthen their loyalty card programs, increase customer engagement, and foster long-term customer loyalty. Simultaneously, researchers can contribute to the advancement of knowledge in this field by further investigating the impact of loyalty card programs on customer behavior and loyalty in diverse settings.

## Data Availability

Figshare: DATASET SRQR CHECKLIST QUESTIONNAIRE IN ENGLISH AND ALBANIAN LANGUAGE. https://doi.org/10.6084/m9.figshare.23397089.v1 ( Brunela and Ervin, 2023) This project contains the following underlying data:
- Dataset Loyalty cards.xlsx Figshare: DATASET SRQR CHECKLIST QUESTIONNAIRE IN ENGLISH AND ALBANIAN LANGUAGE. https://doi.org/10.6084/m9.figshare.23397089.v1 ( Brunela and Ervin, 2023) This project contains the following extended data:
- Questionnaire english and albanian.docx (The questionnaire was designed in albanian language. The translated version in English in done by authors in order to be used as evidence.) Data are available under the terms of the Creative Commons Attribution 4.0 International license (CC-BY 4.0).

## Software availability

Archived source code at the time of publication: https://zenodo.org/record/8163511 ( Ervin and Brunela, 2023)

License: MIT
