# Supplementary material for: Analyzing the impact of loyalty card programs on customer behavior: insights from the Albanian market
Source: F1000Res. 2023 Nov 21;12:1028. Originally published 2023 Aug 23. [Version 3] doi: 10.12688/f1000research.138185.3 (PMC10682604; doi:10.12688/f1000research.138185.3)
Supplement: Peer review history

# Reviewer report: Reviewer response for version 3

**DOI:** 10.5256/f1000research.158974.r224413

**Published:** 2023-11-27

**Authors:** Canan Saricam

**Affiliations:** 1 Textile Engineering, Istanbul Technical University, Istanbul, İstanbul, Turkey

The revised version of the manuscript was reviewed and it was observed that the authors have made sincere efforts in the revised version.

 In this respect, the research gaps in the theory and practice were underlined. 

 Logistic regression was employed in the analyses.

 The unnecessary headings were eliminated.

 Even though the paper has some limitations in terms of theoretical implications, considering the practical implications as well as the improvement in the presentation of the paper, the paper can be published as it is.

Is the work clearly and accurately presented and does it cite the current literature?

Partly

If applicable, is the statistical analysis and its interpretation appropriate?

No

Are all the source data underlying the results available to ensure full reproducibility?

Partly

Is the study design appropriate and is the work technically sound?

Partly

Are the conclusions drawn adequately supported by the results?

Partly

Are sufficient details of methods and analysis provided to allow replication by others?

Partly

Reviewer Expertise:

Textile and Fashion Marketing

I confirm that I have read this submission and believe that I have an appropriate level of expertise to confirm that it is of an acceptable scientific standard.

# Reviewer report: Reviewer response for version 3

**DOI:** 10.5256/f1000research.158974.r224414

**Published:** 2023-11-22

**Authors:** Ali Ismajli

**Affiliations:** 1 AAB College, Prishina, Kosovo (Serbia and Montenegro)

Better

Is the work clearly and accurately presented and does it cite the current literature?

Yes

If applicable, is the statistical analysis and its interpretation appropriate?

I cannot comment. A qualified statistician is required.

Are all the source data underlying the results available to ensure full reproducibility?

Yes

Is the study design appropriate and is the work technically sound?

Yes

Are the conclusions drawn adequately supported by the results?

Yes

Are sufficient details of methods and analysis provided to allow replication by others?

Yes

Reviewer Expertise:

Marketing: marketing management; market research; customer behavior's; Customer satisfaction; customer loyalty

I confirm that I have read this submission and believe that I have an appropriate level of expertise to confirm that it is of an acceptable scientific standard.

# Reviewer report: Reviewer response for version 2

**DOI:** 10.5256/f1000research.157040.r215718

**Published:** 2023-11-01

**Authors:** Canan Saricam

**Affiliations:** 1 Textile Engineering, Istanbul Technical University, Istanbul, İstanbul, Turkey

**Review for Revised version of “Analyzing the impact of loyalty card programs on customer behavior: insights from the Albanian market”**

 The revised version of the manuscript was reviewed and it was observed that the authors have made sincere efforts in the revised version. The manuscript can be considered for indexing, however the originality of the manuscript is still weak and some major changes are required. Below the changes made on the manuscript were reviewed and the points, which still required modification were listed:

- The introduction section has been re-written and more facts were included in regards to Albanian market.
- The literature part was divided into two parts as suggested. Specific references conducted in Albanian market was included.

However, this part still needs a minor modification. I would suggest to underline the research gaps in terms of theory and practice. Limited number of the studies analyzing the relationship between awareness and the other variables (consumer satisfaction and loyalty) might be one research gap and fulfilling the research gap in this regards might provide theoretical implication.  The application of study in Albanian market is related with the scope of the study and having limited number of the study might be another research gap and fulfilling this research gap might provide practical implications. (These points were already mentioned but given in a way that it was a research gap specific to Albanian market, in my opinion the research gaps can be underlined by separating these two points.)

 On the other hand, the originality of the manuscript seems still weak.

 Data analysis: More explanation was given in the data analysis and it was stated that the questionnaire is a custom designed one. The questions should be given in a table.

 The problem about making the analysis by using answers for single question still exists. But the authors applied regression analysis to put forward the relationship between different variables, awareness, satisfaction and loyalty. However, no information was given about the type of the regression analysis applied. If the variables are measured with a single question and categorical variables, logistic regression should be applied.

 Last but not least, there are many subheadings under results, discussion and conclusion parts. The sub-heading in results and discussion parts can be eliminated and they can be reorganized to have lower number of subheadings in conclusion part.

Is the work clearly and accurately presented and does it cite the current literature?

Partly

If applicable, is the statistical analysis and its interpretation appropriate?

No

Are all the source data underlying the results available to ensure full reproducibility?

Partly

Is the study design appropriate and is the work technically sound?

Partly

Are the conclusions drawn adequately supported by the results?

Partly

Are sufficient details of methods and analysis provided to allow replication by others?

Partly

Reviewer Expertise:

Textile and Fashion Marketing

I confirm that I have read this submission and believe that I have an appropriate level of expertise to confirm that it is of an acceptable scientific standard, however I have significant reservations, as outlined above.

# Reviewer report: Reviewer response for version 2

**DOI:** 10.5256/f1000research.157040.r215717

**Published:** 2023-10-19

**Authors:** Ali Ismajli

**Affiliations:** 1 AAB College, Prishina, Kosovo (Serbia and Montenegro)

It appears the author has addressed the reviewers' comments.

Is the work clearly and accurately presented and does it cite the current literature?

Yes

If applicable, is the statistical analysis and its interpretation appropriate?

I cannot comment. A qualified statistician is required.

Are all the source data underlying the results available to ensure full reproducibility?

Yes

Is the study design appropriate and is the work technically sound?

Yes

Are the conclusions drawn adequately supported by the results?

Yes

Are sufficient details of methods and analysis provided to allow replication by others?

Yes

Reviewer Expertise:

Marketing: marketing management; market research; customer behavior's; Customer satisfaction; customer loyalty

I confirm that I have read this submission and believe that I have an appropriate level of expertise to confirm that it is of an acceptable scientific standard.

# Reviewer report: Reviewer response for version 1

**DOI:** 10.5256/f1000research.151369.r200091

**Published:** 2023-09-22

**Authors:** Canan Saricam

**Affiliations:** 1 Textile Engineering, Istanbul Technical University, Istanbul, İstanbul, Turkey

The manuscript analyzes the consumer behavior for a specific market, for this reason, the findings cannot be generalized to provide practical implications. More important than that, the novelty is not good for the manuscript and it does not provide any contribution to literature. Last but not least, the statistical analyzes selected are not convenient for answering the research questions and analyzing the consumer behavior well. For all these reasons mentioned above, I suggest the manuscript is not approved for indexing. Below these points were summarized in much more detail with some suggestions that should be made:

**Introduction:** The introduction (background) part was written in a way that it summarizes the importance of the loyalty cards and the findings of some past studies, which have similar type of research objectives. It is suggested to re-write the introduction part in a way that it underlines the missing points in the literature and explains why this study is needed.

 Moreover, the study was established in a specific market, which prevents to generalize the findings. This is one limitation of this study, but this limitation can be eliminated by giving some specific reasons for this selection. If there is not any, at least the authors should introduce the market, give some facts and cite some studies already established in this market to introduce the consumer base more.

**Literature review:** This part was written under 4 headings and gaps in literature were summarized at the end of the literature section. However, these research gaps were not underlined in these 4 sections. I would suggest to organize the literature part in 2 sections. In the first section, some information might be given about the concept, benefits and incentives of loyalty card problems and; in the second section, the studies about consumer behavior might be summarized for these loyalty card by highlighting the lacking points. Here the number of studies about awareness should also be mentioned. Finally, this section should include and specify the studies established in the Albanian market.

**Data analysis:** The questionnaire, which was designed by the same authors, was used by the authors. Even though, the authors are the same, the design of this questionnaire should be mentioned in this study. This is critical to show the theoretical implications, which are quite missing for this study.

 I understand that, most of the evaluations were made with single question, which can be quite defective. Moreover, chi-square difference tests do not give any idea about the relationship between two variables  (the research question 2 and 3). I suggest to employ regression analyses

 The theoretical implications and practical implication should be added before the limitations in the manuscript.

Is the work clearly and accurately presented and does it cite the current literature?

Partly

If applicable, is the statistical analysis and its interpretation appropriate?

No

Are all the source data underlying the results available to ensure full reproducibility?

Partly

Is the study design appropriate and is the work technically sound?

Partly

Are the conclusions drawn adequately supported by the results?

Partly

Are sufficient details of methods and analysis provided to allow replication by others?

Partly

Reviewer Expertise:

Textile and Fashion Marketing

I confirm that I have read this submission and believe that I have an appropriate level of expertise to state that I do not consider it to be of an acceptable scientific standard, for reasons outlined above.

# Response

**Published:** 2023-10-03

**Authors:** Ervin Myftaraj

**Affiliations:** Marketing, Universiteti Aleksander Moisiu Durres, Durrës, Durrës County, Albania

Dear Reviewer,

 Thank you for you suggestions. I already applied all your suggestion in the version 2 of my manuscript.

 Please check it and let me know if something else needs addressing.

 Thank you very much,

 Ervin

# Reviewer report: Reviewer response for version 1

**DOI:** 10.5256/f1000research.151369.r203861

**Published:** 2023-09-08

**Authors:** Ali Ismajli

**Affiliations:** 1 AAB College, Prishina, Kosovo (Serbia and Montenegro)

The manuscript titled "Analyzing the impact of loyalty card programs on customer behavior: insights from the Albanian market" presents an in-depth investigation into the effects of loyalty card programs on customer behavior within the Albanian retail market. The study employs a well-structured research design, involving a comprehensive survey to assess awareness levels, customer satisfaction, loyalty, and the influence of customized benefits associated with loyalty cards. This peer review evaluates the entire manuscript, encompassing the abstract, introduction, literature review, analysis, findings, conclusions, and recommendations.

**Major Strengths:**

1. **Clear Research Objectives:** The manuscript establishes clear research objectives and hypotheses, providing a solid framework for the study.
2. **Methodological Rigor:** The research methodology is robust and well-detailed, with the survey conducted among a diverse sample of customers in Albania, enhancing its reliability.
3. **Relevance of Findings:** The findings are highly relevant to the retail industry, offering practical insights for enhancing customer engagement and loyalty.
4. **Concise and Well-Structured:** The manuscript is well-organized, making it easy for readers to follow the logical flow of the study.

Given the study's focus on the Albanian market, it is important to acknowledge potential limitations concerning the applicability of the findings to other contexts. The manuscript should thoroughly discuss these limitations and propose avenues for future research or cross-cultural comparisons.

 In the "Discussion" section, where the manuscript briefly mentions that certain loyalty card benefit categories lacked statistically significant associations with loyalty, a more comprehensive examination of these non-significant findings and exploration of potential contributing factors would augment the paper's comprehensiveness.

**Recommendation:**

 This study has the potential to make a valuable contribution to the field of marketing and customer behavior. The research findings offer practical implications for retailers and marketers aiming to enhance customer loyalty through loyalty card programs. Therefore, I recommend approving the manuscript for indexing.

Is the work clearly and accurately presented and does it cite the current literature?

Yes

If applicable, is the statistical analysis and its interpretation appropriate?

I cannot comment. A qualified statistician is required.

Are all the source data underlying the results available to ensure full reproducibility?

Yes

Is the study design appropriate and is the work technically sound?

Yes

Are the conclusions drawn adequately supported by the results?

Yes

Are sufficient details of methods and analysis provided to allow replication by others?

Yes

Reviewer Expertise:

Marketing: marketing management; market research; customer behavior's; Customer satisfaction; customer loyalty

I confirm that I have read this submission and believe that I have an appropriate level of expertise to confirm that it is of an acceptable scientific standard.
